# The Case of Brown

**Published:** 1878-07

**Authors:** Ben. H. Pratt


### THE BISTOURY.

Vol. XIV. ELMIRA, N. Y., JULY, 1878. No. 2.

### Contributions.

### THE CASE OF BROWN.

BEN H. PRATT.

Brown, until he was thirty odd, considered himself about the wholesomest man in town. His height, weight, breadth and solidity, were such as to attract attention and beget envy. He never realized what ailments were, he could not comprehend them. He had the usual amount of sympathy for the ill, and used the conventional phrases in expressing his condolence with, and sorrow for, those of his friends who were suffering from disease, or were temporarily indisposed under the countless ills which afflict the great majority of the human family. Headaches, rheumatisms, dyspepsia—victims to which he met every day, in business, in society, on the street, at table—had become quite familiar to him, theoretically. He had heard enough about these to be clear in his own mind that he would readily recognize any of them were he to be attacked thereby. And yet he maintained that many, if not all the ailments to which men and women were heir, were the resultants of excesses, indiscretions, imprudences and violations of the laws of health. Because he was not an ailer himself, he believed he owed his physical validity to his own prudence, and yet he could never clearly explain how his habits differed in any material particular from those of others with whom he associated. Still he prided himself, not boastfully, however, upon his excellent health, and with a manner that implied blame on the part of every invalid. Very well people do this quite generally, seemingly holding to that hygienic heterodoxy which proclaims all men created physically equal, which is as great a fallacy as that one which avers that all men are created free and equal politically—a little misapprehension on the part of our governmental framers, which we wink at to this day, but don't believe—not a man of us. Brown felt a peculiar pity for the sick and complaining, not so much, perhaps, because of any suffering they endured, for he couldn't really know what that was; but because they were deprived of so much of the enjoyment of life; and what was still more patent to him, often deprived him of many enjoyments. They could not enter with him into his healthful appreciation of everything; they rather begloomed little portions of his life; they often bored him, after the manner of the average invalid, with synopses of their cases—themes which rarely interest those who have never experienced the ills detailed. There are very few invalids, however fascinating their descriptive powers may be, who can make a narrative of the rise and progress of their invalidism anything akin to an enjoyable revelation; in fact, to be plain about it, such detail is usually a bore of the first quality, and none enjoy being bored unless, like the doctors, they are pretty well paid for it. So Brown became a little impatient with even his very good friends, and by and by he avoided those who had a habit of being periodically or occasionally sick, lest perchance they should inflict upon him the old, old story of how it all happened, and just how they endured it all.

Now it came to pass, one morning, in the life of our friend Brown, that he got up feeling queer. His usually clear head felt heavy; there was an unpleasant taste in his mouth; as he was taking his ablutions he caught sight of his eyes in the glass, and they were far from clear; his stomach didn't yearn for breakfast as formerly, but on the contrary seemed to have adopted the theory that it would be much better to give than to receive, just then. This was all a new experience for Brown. He began to wonder if he was sick. He thought of all he had eaten during the past week—where he had been, and what he had done, that could be construed into a violation of the laws of health as he had always understood them—finally concluded to say nothing about it, and diet a day or two—ate no breakfast—went to business—worried through the day very miserably—pleaded outside lunch as excuse for a very light dinner, and excessive sleepiness as excuse for going to bed early. He rose better—the next day he was almost entirely his former self, but he carried the air of one who had had an experience the nature of which he is unwilling to share with any. He had lost confidence in himself—he had been so near sickness that he doubted immunity from that which he had begun to believe himself exempt. Weeks passed, and Brown, the usually cheery, volatile, beaming Brown, became much modified as to these characteristics. The change was noted, and he was rallied upon it, when he evasively and ingeniously directed inquiry into other channels or hinted at business perplexities as the cause of the phenomena. But by and by that which was a mere hint of his hygienic derangement assumed a more serious phase. Brown, in his ignorance of disease from personal experience, and yet under a settled conviction that he knew disease when he found it, felt that he could diagnose a case pretty well for a non-professional, and locate with certainty the organ affected; and so, when one day he was suddenly seized with a sense of suffocation—a fearful goneness, as it were—a feeling that his inhalations were not sufficient to vitalize his blood—that the air he breathed was deprived of its ozonizing qualities—that unless something very unusual happened within the next ten minutes, he would be found a cold cadaver—when, we say, he experienced the above, although the recovery was almost as sudden as the attack, was he excusably a much scared man. He who had formerly congratulated himself upon his free dom from the minor ills which humanity, with few exceptions, accepted as conditions of existence, had suddenly been brought face to face with a terror. Death, he firmly believed, had stalked right into his presence and had rattled his bones before his very eyes—had held up a fleshless finger of warning, then given him a poke in the ribs, and left with the air of one who might call again at any moment and complete his usual errand. But Brown wasn't one of those men who impulsively rush for a doctor before they know what to say to him when they find him, but rather one of those who, having thoroughly diagnosed and determined his own case, goes to his physician to have it confirmed. Brown had made up his mind that his was a lung trouble—he didn't know just what sort of a pulmonary difficulty it was, but as to the organ affected he was confident there could be no mistake. His doctor, one of those who, having an intelligent patient, and one so well informed and well balanced as Brown was, are not inclined to dispute his convictions, particularly when there's a right smart chance of losing a patient and a fee by setting up a counter opinion, adopted Brown's diagnosis and advised accordingly. Weeks having passed, and no repetition of the attack having occurred, the correctness of the opinion of Brown, corroborated by that of the doctor, seemed verified, and the patient, though to all appearances as well a man as ever, was by no means as happy an one. He believed himself about to become a victim, in the more or less remote future, of a fatal malady, and the whole tenor of his being was affected by dwelling upon the subject. That he, to whom, until now, disease was so utter a stranger, should, of all his complaining associates, be singled out in this way, was something beyond his comprehension, but he accepted the inevitable and awaited it with resignation. Meantime his usual health was apparently restored, but, as he believed, it was only the dallying of the insidious foe of life, and he ceased not to brood upon what might—what doubtless must happen. Months wore on, and Brown was beginning to wonder if perhaps his attack might not have been less serious than he imagined, and he had nearly concluded that after all he was more scared than hurt, when, as suddenly as before, his system experienced another shock, and this time its prominent feature was, in connection with the suffocating sensation, temporary cessations of the heart's pulsations, followed by heavy and quick throbs of that organ, and accompanied with complete nervous prostration for the time being. If the first attack frightened him, this filled him with untold terror. Again he diagnosed his own case, arrived at the conclusion that another and a still more vital organ was the seat of disease, and that he was liable at any moment to be fatally stricken down. His physician, as before, accepted the theory indicated by the patient, but conveyed the comforting assurance that if the latter were careful to refrain from any undue excitement, and took such care of himself as his medical adviser prescribed, he might live a number of years, even with an affection of the heart. The doctor knew of hundreds of cases in which men had baffled this disease until old age, and then had died of something else. Of course that isn't a pleasant sort of life in which one is debarred from indulgence in all those stimulating and exalting vital activities of existence to which one owes so much of the joys of life—obliged to curb his indignation at every turn, lest he may yield to a commendable desire to kick somebody—but it was better than the alternative, and so Brown accepted it. Probably it was owing to his implicit compliance with his physician's instructions that he never had another attack of the kind referred to, but so it was, and ere many months had elapsed, this too was looked back upon with somewhat of doubt as to its being of the serious character which both doctor and patient had determined it. Indeed these two attacks were, after a time, somewhat eclipsed by another experience of Brown's, which though it did not come in the sudden and shocking way which had characterized the others, was even more dreadful because of the duration of the trouble. This time there came a sense of a terrible weight upon that portion of his internality which seemed to Brown to be directly in the region of the heart, and this was alternated by a still more terrifying sensation, to-wit: a feeling of enlargement of that organ, even to a fear of its bursting asunder unless relief were speedily obtained. The continuous and prostrative nature of this attack prevented Brown from calling upon his own physician, or even summoning him, and the nearest doctor was called. It was an emergency, and there was no time to consult about or discuss the merits of the several medical exponents of the locality. The nearest doctor happened to be one of those who never allowed his patients to do their own diagnosing. If they insisted, he insisted upon their doing the balance of the job, to-wit: their own treatment. There are a few such doctors, and it is a comfort to know that they are becoming more numerous. The consequence, in Brown's case, was, that he was not allowed to get further than a detailment of his symptoms, when the doctor's wallet was out and the medicine administered. Relief from the terrible oppression was so speedily brought about, that the chagrin of Brown at not being allowed to inform the doctor as to what ailed him, gave way to a more than usual confidence in this new physician, who seemed to have intuitively fathomed the whole matter, and, what was still better, had rather pooh-pooed any intimation of anything at all serious in this attack. In the course of the after chat between patient and doctor, Brown naturally reverted to his former troubles, his "pulmonary attack," and his more serious "heart difficulty," and desired to know to which of the two this last was a sequel. The doctor, having listened to the details of both, could not repress a smile at the very lugubrious manner in which they were related, and when the part the doctor first consulted had played in the affair was set forth, the smile became broader and broader until it terminated in a hearty laugh. Brown's surprise at the effect of his narrative upon the doctor, was natural, and an inquiry into the cause followed as a matter of course. The physician then explained the peculiar forms which that very variable disease, dyspepsia, takes under certain circumstances; how that there is scarcely an organ in the thoracic or abdominal cavities that may not be mistakenly fixed upon as the seat of disease when one has a genuine dyspepsia—how frequently patients, and even physicians themselves, are baffled by the multiplicity and wonderful variety of effects from this single cause—how men with lungs intact are made to believe themselves consumptives, and other men without the slightest heart derangement, are for years made to stand in mortal fear of sudden death from a cessation of the circulation—how possible it is for dyspepsia to take the form of almost every known malady and counterfeit it so closely as to deceive the most acute practitioners. Here was a case in point. Brown had given himself over to the consumptives—then to those who go about with the unenviable conviction hanging over them, that some day they will drop down and be carried in somewhere. His life for some years was rendered a burden because of somebody's inability to diagnose a case of dyspepsia. There are thousands to-day suffering from what they suppose to be consumption, or heart disease, or liver complaint, or kidney difficulties, and have given themselves over to life-long invalidism, simply because they do not know, or will not believe, that their whole trouble is dyspepsia, the most prevalent of all maladies because so frequently unrecognized, and when recognized demands too great a self-denial on the part of the patient whose stomach is too often the only shrine he worships at. Yet there are those who would be glad to know that what they deem a mortal disease may be only an aggravated case of dyspepsia, and for such we present the case of Brown, hoping that, like Brown, they may find a sensible and independent doctor, as well as a load lifted off their minds, and above all, that they may learn how dangerous a thing it may be to diagnose one's own case, and insist upon the acceptance, by the doctor, of that diagnosis, which has for its foundation the mere opinion of the patient who is too apt to confound effects and causes, and thereby deceive a too easily convinced physician, or one who is too apt to adapt his treatment to his patient's suggestions and wrongly founded convictions.

—Bound copies of the BISTOURY, for past three years, can be had at this office, for $2.50.

